# Mantle recipes for seafloor tectonics

**DOI:** 10.1093/nsr/nwaf412

**Published:** 2025-09-26

**Authors:** Daniele Brunelli

**Affiliations:** Dipartimento di Scienze Chimiche e Geologiche, Università di Modena e Reggio Emilia, Italy

From a geological perspective, the Earth’s outer shell—the lithosphere—is split into two contrasting domains: continents and oceans. Continents are thick, buoyant and remarkably stable rafts that have floated on the viscous mantle for billions of years. In contrast, oceanic lithosphere has a far more transient life. It forms continuously at mid-ocean ridges, spreads away from them, and is eventually swallowed back into the mantle at subduction zones. This rapid tectonic recycling ensures that oceanic lithosphere rarely survives more than a couple of hundred million years. As a result, nearly two-thirds of Earth’s surface is perpetually renewed, giving the planet a fresh and dynamic skin.

This renewal is assisted by mantle partial melting beneath the ridges, which produces the distinctive mid-ocean ridge basalts (MORBs). Their composition reflects the chemical and lithological makeup of the mantle source, a triangular melting region extending no deeper than about 100 km beneath the ridge axis. The interplay between total melt production and tectonics of the extending ridge defines the morphological appearance of the seafloor. For decades, the prevailing view was that the style of crustal accretion and the resulting morphology depended mainly on the rate of seafloor spreading and the volume of magma supplied, yet seafloor observations tell a more complicated story. Even at a constant spreading rate within a single ridge segment, the mode of spreading can vary dramatically over just a few million years. The associated fluctuations in magma supply would necessitate unrealistic fluctuations of mantle temperature.

An international team led by Chuan-Zhou Liu of the Laoshan Laboratory [1] has now shown that these variations are closely tied to the lithological composition of the mantle beneath the ridge. Their study focuses on the 23°N segment of the Mid-Atlantic Ridge (MAR), a region made famous by the Kane megamullion, the first and best-studied example of long-lived detachment faulting developed during the formation of oceanic core complexes [2]. By linking mantle composition to spreading style, the team provides evidence that the mantle is far from homogeneous.

The upper mantle is, in fact, a geological mosaic. Plate tectonics itself is the

ultimate source of this heterogeneity. As oceanic crust ages, cools, hydrates and collects sediments, it is subducted back into the mantle, while portions of the mantle beneath ridges are progressively depleted in major and trace elements as they melt. Over Earth’s 4.5-billion-year history, these processes have produced a constantly evolving patchwork of rock types: unmelted peridotites, strongly depleted residua and fragments of recycled oceanic crust. The global mantle flow stretches, mixes and reassembles these components into intricate lithological assemblages that eventually rise to partially melt beneath spreading centers.

Most models of MORB generation, however, assume a simplified ‘depleted mantle’ (DM) source. Such models overlook the fine-scale heterogeneity of real mantle mixtures. Melting a composite source introduces important thermal effects, because lithologies with different melting points diffuse heat to one another [3]. This process is controlled by the relative amount and fertility of different lithological components.

In this issue, Zhang *et al.* (2025) addressed this complexity by comparing osmium and neodymium isotopes in parent mantle peridotites and associated basalts, respectively [1]. Their analyses revealed temporal changes in the mantle ‘recipe’ beneath the studied ridge segment. During the long phase of asymmetric extension leading to the formation of the Kane megamullion, the subridge mantle consisted of a mixture of fertile mantle peridotites called lherzolite and strongly depleted peridotites, the latter having lost incompatible elements during earlier melting events. This depletion modifies parent/daughter ratios in radiogenic element systems, which over billion-year decay times develop chemical signatures attesting to ancient depletion events [4].

**Figure 1.**
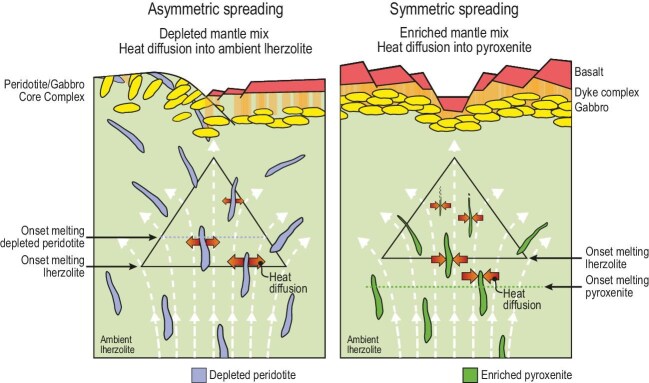
Changing mantle lithological recipes invert the direction of heat diffusion in the subridge melting region, controlling melt production and spreading style. Left: a depleted mantle mix. High-solidus depleted peridotites (blue) dispersed in ambient lherzolite (pale green) diffuse heat into the surrounding lherzolite. They survive partial melting and contribute little to extracted melt, reducing melt productivity and promoting asymmetric spreading with core complex formation. Right: an enriched mantle mix. Low-solidus pyroxenites (green) draw heat from ambient lherzolite, enhancing melting until consumed. This boosts melt productivity, thickens crust and favors symmetric spreading [1].

Around 3 million years ago, the spreading mode at the 23°N MAR segment shifted from asymmetric detachment faulting to symmetric graben-like spreading. This morphotectonic change coincided with both a shift in mantle chemistry and an abrupt increase in total crustal thickness, indicating that the mantle recipe has changed to an enriched mix made by more fertile lithologies (pyroxenites) dispersed in the ambient lherzolite. Changing the mantle lithological recipe inverts the direction of heat diffusion: in a depleted mantle mix, heat diffuses from high-solidus depleted peridotites into ambient lherzolites, while it diffuses from ambient lherzolites into low-solidus pyroxenites when an enriched mantle mix arrives (Fig. 1), boosting the partial melting of the enclosed pyroxenites [5]. The differential heat transfer versus melt productivity between depleted and enriched components drives the total melt production (crustal thickness) and its chemical composition. Modeling the magma fluxes for the different mantle assemblages confirmed these observations.

The implications are profound. The work of Zhang *et al.* [1], shows that short (kilometer-scale) lithological contrasts dictate the tectonic style and magmatic budget of ridge segments. The classical view of a uniform depleted mantle must give way to a picture of the mantle as a fine-grained aggregate of diverse lithologies, each with distinct chemical and isotopic fingerprints. Such small-scale differences affect mantle rheology, influencing how it flows and deforms.

The challenge now is to map this elusive heterogeneity along ridge systems worldwide. Results from Zhang *et al.* [1] suggest that different ridge regions host distinct lithological mixtures. Characterizing these ‘recipes’ of the mantle will be a powerful tool for reconstructing the deep history of our planet. More broadly, this research emphasizes that the mantle is not a silent background actor but an active driver of the ever-changing face of Earth’s surface.
